# Development and validation of a risk nomogram for postoperative acute kidney injury in older patients undergoing liver resection: a pilot study

**DOI:** 10.1186/s12871-022-01566-z

**Published:** 2022-01-13

**Authors:** Yao Yu, Changsheng Zhang, Faqiang Zhang, Chang Liu, Hao Li, Jingsheng Lou, Zhipeng Xu, Yanhong Liu, Jiangbei Cao, Weidong Mi

**Affiliations:** 1 Medical School of Chinese PLA, 28th Fuxing Road, Haidian District, Beijing, 100853 China; 2 Department of Anesthesiology, The First Medical Center of Chinese PLA General Hospital, 28th Fuxing Road, Haidian District, Beijing, 100853 China; 3 Medical College of Nankai University, 94th Weijin Road, Nankai District, Tianjin, 300074 China

**Keywords:** Acute kidney injury, Hepatectomy, Elderly patients, Renal injury, Risk score, Prediction

## Abstract

**Background:**

Postoperative acute kidney injury (AKI) is associated with poor clinical outcomes. Early identification of high-risk patients of developing postoperative AKI can optimize perioperative renal management and facilitate patient survival. The present study aims to develop and validate a nomogram to predict postoperative AKI after liver resection in older patients.

**Methods:**

A retrospective observational study was conducted involving data from 843 older patients scheduled for liver resection at a single tertiary high caseload general hospital between 2012 and 2019. The data were randomly divided into training (70%, *n* = 599) and validation (30%, *n* = 244) datasets. The training cohort was used to construct a predictive nomogram for postoperative AKI with the logistic regression model which was confirmed by a validation cohort. The model was evaluated by receiver operating characteristic (ROC) curve, calibration plot, and decision curve analysis in the validation cohort. A summary risk score was also constructed for identifying postoperative AKI patients.

**Results:**

Postoperative AKI occurred in 155 (18.4%) patients and was highly associated with in-hospital mortality (5.2% vs. 0.7%, *P* <  0.001). The six predictors selected and assembled into the nomogram included age, preexisting chronic kidney disease (CKD), non-steroidal anti-inflammatory drugs (NSAIDs) usage, intraoperative hepatic inflow occlusion, blood loss, and transfusion. The predictive nomogram performed well in terms of discrimination with area under ROC curve (AUC) in training (0.73, 95% confidence interval (CI): 0.68–0.78) and validation (0.71, 95% CI: 0.63–0.80) datasets. The nomogram was well-calibrated with the Hosmer-Lemeshow chi-square value of 9.68 (*P* = 0.47). Decision curve analysis demonstrated a significant clinical benefit. The summary risk score calculated as the sum of points from the six variables (one point for each variable) performed as well as the nomogram in identifying the risk of AKI (AUC 0.71, 95% CI: 0.66–0.76).

**Conclusion:**

This nomogram and summary risk score accurately predicted postoperative AKI using six clinically accessible variables, with potential application in facilitating the optimized perioperative renal management in older patients undergoing liver resection.

**Trial registration:**

NCT04922866, retrospectively registered on clinicaltrials.gov on June 11, 2021.

**Supplementary Information:**

The online version contains supplementary material available at 10.1186/s12871-022-01566-z.

## Background

Despite the recent technical advances in surgical procedures, there have been considerably high rates of operative mortality and morbidity resulting from complex surgical procedures, especially in emergency surgery cases [1]. Among the various types of postoperative organ injuries, acute kidney injury (AKI) is particularly prevalent in 5 to 20% of patients undergoing major non-cardiac surgery and in 10 to 40% of high-risk patients with hepatic resection surgery [2–5]. Previous studies have confirmed AKI as an independent contributor to peri- and postoperative morbidity and mortality in both cardiac and non-cardiac surgeries [6–9]. It has been reported that postoperative AKI could increase the risk of death by twelve folds and extend the hospital stay by five days than usual [2], and may also contribute to the development of advanced chronic kidney disease (CKD), involving substantial healthcare burden [10].

In the older population, however, the incidence of postoperative AKI is significantly higher as the filtration capacity of the kidney decreases about 1% every year after the age of 40, even in the healthy population [11, 12]. Microstructural and functional changes in the kidney-related to healthy aging are reportedly aggravated in the presence of CKD [11]. Aging reduces renal autoregulatory capacity due to physiological and functional changes, leading to different types of kidney diseases, such as vascular sclerosis [13], declining glomerular filtration rate (GFR) [14], thereby enhancing the susceptibility of the older population to postoperative AKI. Notably, Chao et al found that almost 20% of patients in a cohort of 4000 older subjects developed postoperative AKI [15]. Moreover, postoperative AKI cases can significantly increase during certain procedures, such as cardiac and vascular surgeries, up to 30% [16]. In contrast to cardiac or vascular surgery-associated postoperative AKI incidence, there are not enough studies on hepatic resection-related AKI onset and its risk factors and prognosis.

Generally, in-depth knowledge of risk factors and identification of high-risk populations, who are predisposed to develop AKI is urgently warranted as a preventive measure to minimize operative mortality rate and to design efficient therapeutic strategies. Efficient preventive measures to reduce the risk of AKI include precise assessment of renal functions, careful administration of nephrotoxic drugs, minimizing procedural injuries, and an effective intravenous fluid regimen. However, no validated model is currently available to predict the risk of postoperative AKI in older patients following liver resection surgery. Therefore, the present study aimed to establish and validate a predictive nomogram and a simple risk score assessment to identify the risk of developing postoperative AKI in older patients following liver resection. It would be of great clinical significance for clinicians to be able to predict whether a patient would need special attention for optimal renal management after hepatectomy surgery.

## Methods

This retrospective observational study was conducted using the dataset of inpatient surgeries in the First Medical Center of Chinese PLA General Hospital. The study design and data analysis were approved by the Institutional Committee for Medical Research Ethics (approval number: S2021–335-01) and registered at clinicaltrials.gov (identifier: NCT04922866). The requirement for written informed consent was waived for this retrospective study. This manuscript adheres to the *Transparent Reporting of a Multivariable Prediction Model for Individual Prognosis or Diagnosis (TRIPOD)* guidelines [17].

### Patients

Older patients (≥ 65 years) admitted to the hospital for elective hepatectomy between January 2012, and July 2019 were included in this cohort. While patients no more than 65 years old, preoperative baseline GFR < 15 ml·min^− 1^·1.73 m^− 2^, emergency operation, concurrent operation, laparoscopic operation, or liver transplantation surgery were excluded.

### Data collection

Data acquisition from the electronic medical record (EMR) system was performed using SQL Server (Microsoft, USA). To facilitate the clinical use of predictors, we considered the inclusion of both preoperative and intraoperative parameters into the nomogram, which might closely affect renal function. From the patient record integrated management system (PRIDE 2.1.2.193, Heren Health, China), we extracted the relevant patient demographics, including age, sex, body mass index (BMI), combined hypertension, diabetes, cardiovascular and pulmonary diseases, CKD, American Society of Anesthesiologists physical score (ASA PS), and length of hospital stay. The prescribed medication regimen included non-steroidal anti-inflammatory drugs (NSAIDs), nephrotoxic antibiotics, glucocorticoids, and preoperative and intraoperative diuretics. Serum biomarkers included serum creatinine, albumin, bilirubin, hemoglobin, and fasting blood glucose. From the anesthesia information management systems (DoCare 3.1.0 build 153, MEDICALSYSTEM, China), intraoperative data were retrieved, including types and duration of the surgical procedures performed and extent of liver resection, intraoperative hepatic inflow occlusion, blood pressure and infusion of vasoactive drugs, the requirement for intraoperative infusion and transfusion, and volumes of blood loss and urine output.

### Definitions of outcomes

The primary outcome was postoperative AKI, defined as an absolute increase in serum creatinine level of ≥26.5 μmol·l^− 1^ within 48 h or a 1.5-fold increase from preoperative baseline within seven days after surgery, according to the *Kidney Disease: Improving Global Outcomes (KDIGO)* criterion [18]. Baseline creatinine or eGFR were defined as the closest measurement before the date of operation, within 3 months. Related definitions and diagnoses included the following: hypertension defined as systolic blood pressure ≥ 140 mmHg and/or diastolic blood pressure ≥ 90 mmHg on three different occasions; diabetes defined as reported history or physician-diagnosed diabetes or the presence of antidiabetic medication; cardiovascular diseases included reported history or physicians-diagnosis of coronary heart disease, angina, myocardial infarction, heart failure, or stroke; pulmonary disease defined as self-reported chronic bronchitis, chronic obstructive pulmonary disease (COPD), emphysema, interstitial lung disease (ILD), and asthma; preoperative CKD was defined as the GFR of less than 60 ml·min^− 1^·1.73 m^− 2^ for adults based on the CKD Epidemiology Collaboration (CKD-EPI) equation [19]. Blood transfusion was defined as any allogeneic transfusion of at least a single unit of red blood cells.

### Statistical analysis

Continuous variables were reported as medians with quartiles and compared using Wilcoxon rank-sum test as they were abnormally distributed checked by Skewness-Kurtosis All test. Categorical data were reported as frequencies and percentages and compared using the chi-squared test or Fisher’s exact test as appropriate.

The overall 843 patients were divided into training (*n* = 599) and validation (*n* = 244) cohorts with a split ratio of 70 and 30% using randomization method. The training dataset was used to develop the prediction model in the final logistic regression. Firstly, a univariate analysis was performed using the Wilcoxon rank-sum test for continuous variables and the chi-squared test for categorical variables, with postoperative AKI as the outcome. Secondly, for the independent predictors included in the model, candidate variables that were clinically relevant to AKI and *P*-value less than 0.1 in univariate analysis were included in the multivariable model. Additionally, the number of outcome events was considered, that is, at least ten outcome events per variable (EPV) generally [20]. Based on the EPV approach for determining sample size, our sample size could be expected to provide robust estimates. Thirdly, the enter methodology was applied to select predictive variables in the final model. Finally, the multivariable logistic regression model was formulated to establish the prediction model, and a nomogram was further performed to predict postoperative AKI in the training dataset.

The predictive performance of the model was subsequently evaluated in patients from the validation cohort. The discrimination of the nomogram was assessed by calculating the area under the receiver operating characteristic (ROC) curve. The model’s calibration was evaluated using the Hosmer-Lemeshow goodness of fit test. Finally, the decision curve analysis (DCA) was performed to reveal the net benefits with each threshold probability [21]. Statistical analyses were performed using R 4.0.1 (R Foundation for Statistical Computing, Vienna, Austria) and SPSS 25.0 (IBM Corp., Chicago, IL, USA) software. Statistical significance was accepted at the 0.05 level, and all tests were two-tailed.

## Results

### Patient characteristics

**Fig. 1:**
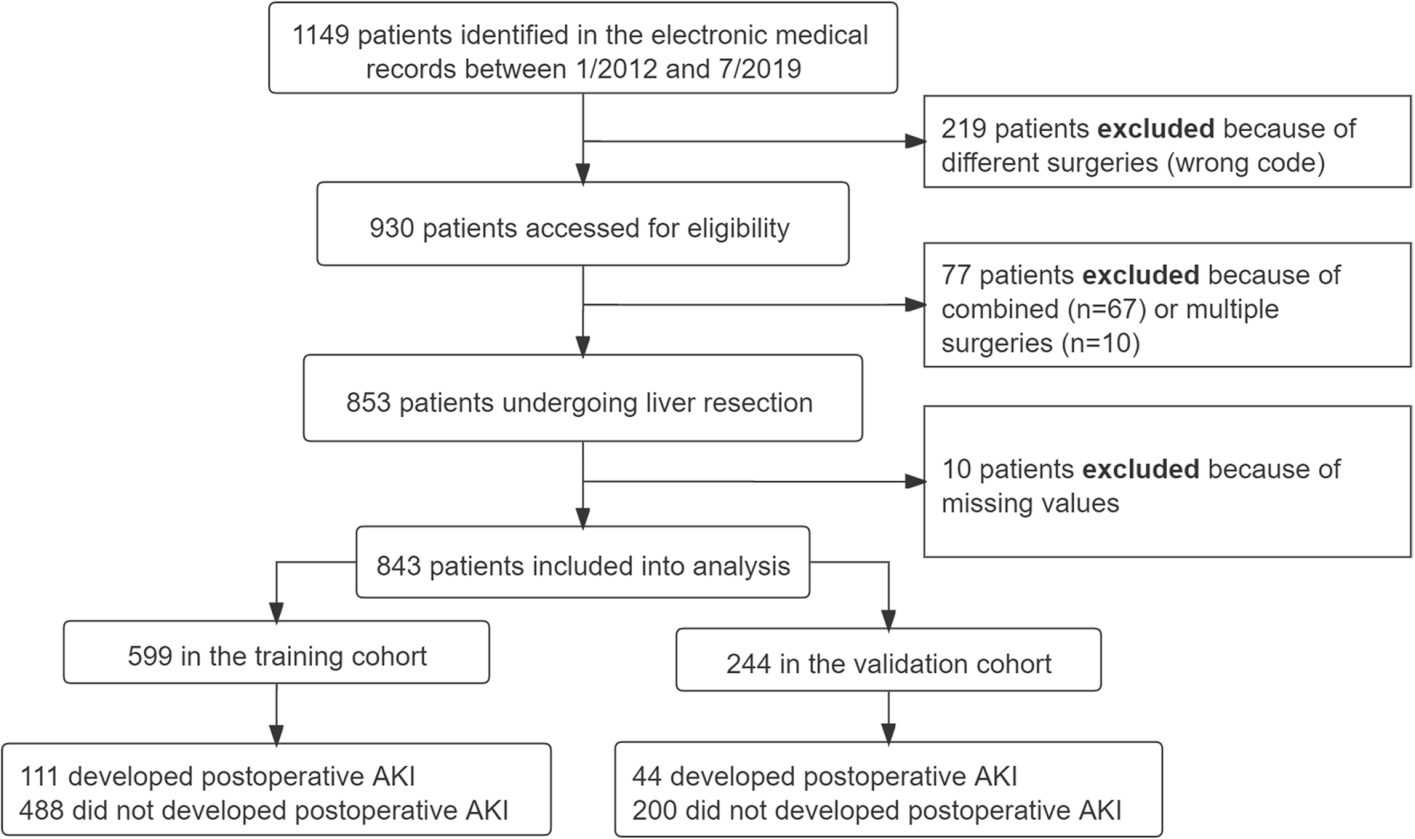
Study population enrolled and outcomes in the training and validation datasets. Abbreviations: AKI, Acute kidney injury

A total of 930 participants were screened for eligibility from January 2012 to July 2019. After the exclusion of 77 patients who undertook combined or multiple surgeries, 853 patients were selected with liver resection. Additionally, ten subjects with missing values were excluded, thus leaving behind 843 individuals who were enrolled in the final analysis (Fig. 1). Patients were divided into training (599 patients total, 111 postoperative AKI) and validation (244 patients total, 44 postoperative AKI) cohorts, respectively. Patient characteristics and perioperative variables for the overall population are listed in Tables 1 and 2. The median (quartile) age at the time of surgery was 69 (66, 73) years, where 553 (66%) patients were male, and 290 (34%) patients were female. The median (quartile) duration of operation was 3.4 (2.6, 4.5) h, and the median time to discharge was 10 (8, 13) days. Thirteen (1.5%) patients died before discharge.

**Table 1:** Patient Characteristics and baseline variables stratified by datasets

Variables	Training (***n*** = 599)	Validation (***n*** = 244)	***P*** Value
Age, years	69 (66, 72)	69 (66, 73)	0.36
Sex (Male), n (%)	385 (64.3)	168 (68.9)	0.23
BMI, kg·m^− 2^	23.6 (21.6, 25.7)	23.5 (20.9, 26.0)	0.68
Hypertension, n (%)	230 (38.4)	89 (36.5)	0.64
Diabetes, n (%)	109 (18.2)	48 (19.7)	0.63
Cardiovascular diseases, n (%)	80 (13.4)	43 (17.6)	0.13
CKD, n (%)	26 (4.3)	6 (2.5)	0.24
Pulmonary diseases, n (%)	32 (5.3)	12 (4.8)	0.87
Hepatitis/ cirrhosis, n (%)	125 (20.9)	49 (20.1)	0.85
ASA PS, n (%)			0.77
I-II	491 (82.0)	203 (83.2)	
III-IV	108 (18.0)	41 (16.8)	
Pathology, n (%)			0.13
Hepatoma	383 (63.9)	154 (63.1)	
Cholangiocarcinoma	82 (13.7)	46 (18.9)	
Hepatic Metastasis	16 (2.7)	8 (3.3)	
Benign	118 (19.7)	36 (14.8)	
Hemoglobin, g·L^− 1^	131 (120, 142)	130 (119, 140)	0.34
Albumin, g·L^− 1^	39.0 (36.1, 41.6)	39.0 (36.8, 41.4)	0.71
Total bilirubin, μmol·L^−1^	13.1 (9.2, 19.6)	12.6 (9.7, 19.7)	1.00
Direct bilirubin, μmol·L^−1^	4.3 (3.0, 7.5)	4.3 (3.0, 7.7)	0.90
Fasting blood glucose, μmol·L^−1^	5.04 (4.58, 5.83)	5.12 (4.62, 5.96)	0.51
Creatinine, μmol·L^−1^	68.4 (59.0, 79.2)	66.1 (58.5, 76.8)	0.29
eGFR, ml·min·1.73 m^−2^	89.8 (80.8, 94.4)	90.1 (84.5, 95.2)	0.11

Notes: Continuous data are shown as medians (quartiles) and compared using Wilcoxon rank-sum test. Categorical variables are shown as frequencies (percentages) and compared using the chi-squared test or Fisher’s exact test as appropriate

*Abbreviations*: *ASA PS* American Society of Anesthesiologists physical score, *BMI* Body mass index, *CKD* Chronic kidney disease; eGFR, estimated glomerular filtration rate

**Table 2:** Pre- and intraoperative variables and outcomes stratified by datasets

Variables	Training (n = 599)	Validation (n = 244)	***P*** Value
NSAIDs, n (%)	540 (90.2)	217 (88.9)	0.62
Preoperative diuretics, n (%)	41 (6.8)	19 (7.8)	0.66
Nephrotoxic antibiotics, n (%)	52 (8.7)	27 (11.1)	0.30
Glucocorticoid, n (%)			0.75
Dexamethasone	163 (27.2)	71 (29.1)	
Methylprednisolone	277 (46.2)	106 (43.4)	
None	159 (26.5)	67 (27.5)	
Vasoactive agents, n (%)			0.14
None	326 (54.4)	122 (50.0)	
Hypertensive agents	166 (27.7)	79 (32.4)	
Hypotensive agents	62 (10.4)	32 (13.1)	
Both	45 (7.5)	11 (4.5)	
Intraoperative diuretics, n (%)	90 (15.0)	48 (19.7)	0.10
MAP < 60 mmHg, n (%)	378 (63.1)	169 (69.3)	0.10
Duration of MAP < 60 mmHg, min	5 (0, 20)	10 (0, 20)	0.08
Duration of operation, h	3.4 (2.7, 4.5)	3.4 (2.6, 4.7)	0.95
Fluid balance, ml· kg^− 1^· h^− 1^	11.8 (9.0, 14.9)	11.5 (9.0, 14.6)	0.77
Hydroxyethyl starch, ml· kg^−1^· h^− 1^	3.6 (2.6, 4.9)	3.6 (2.7, 4.9)	0.89
Ringer’s solution, ml· kg^− 1^· h^− 1^	9.9 (7.7, 12.9)	9.8 (7.5, 12.5)	0.46
Urine output, ml· kg^− 1^· h^− 1^	1.7 (1.0, 2.8)	1.5 (0.9, 2.8)	0.33
Blood loss, 100 ml	3 (2, 6)	3 (2, 5)	0.99
Blood transfusion, n (%)	137 (22.9)	55 (22.5)	1.00
Resection extent, n (%)			0.55
Right liver	86 (14.4)	34 (13.9)	
Left liver	155 (25.9)	72 (29.5)	
Partial	358 (59.8)	138 (56.6)	
Hepatic inflow occlusion, n (%)	362 (60.4)	151 (61.9)	0.76
Duration of occlusion, min	14 (0, 30)	14 (0, 27)	0.82
LOS, days	10 (8,13)	10 (8,13)	0.79
Postoperative AKI, n (%)	111 (18.5)	44 (18.0)	1.92
Death before discharge, n (%)	7 (1.2)	6 (2.5)	0.22

**Notes:** “Hypertensive agents” in vasoactive agents include ephedrine, epinephrine, dopamine, norepinephrine, and phenylephrine. “Hypotensive agents” in vasoactive agents include urapidil and nicardipine. Nephrotoxic antibiotics refer to aminoglycoside and sulfonamide antibiotics. Continuous data are shown as medians (quartiles) and compared using Wilcoxon rank-sum test. Categorical variables are shown as frequencies (percentages) and compared using the chi-squared test or Fisher’s exact test as appropriate

*Abbreviations*: *AKI* Acute kidney injury, *LOS* Length of hospital stay, *MAP* Mean arterial pressure, *NSAIDs* Non-steroidal anti-inflammatory drugs

Overall, 155 (18.4%) patients developed postoperative AKI. Patients who developed postoperative AKI were at a higher risk of death (5.2%) before discharge than those without AKI (0.7%, *P* <  0.001) (Additional file 1).

### Development of a predictive nomogram

**Fig. 2:**
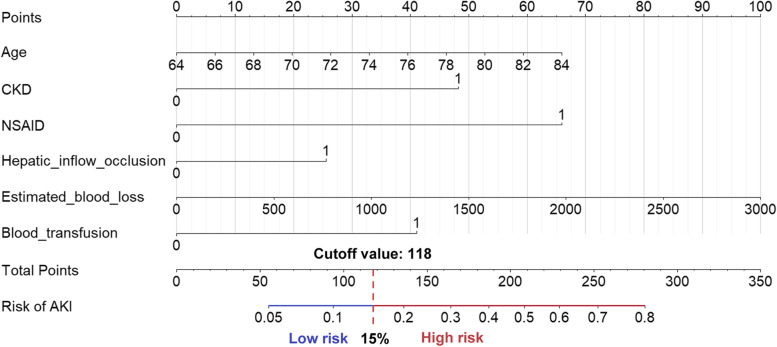
Development of a nomogram for predicting the probability of postoperative AKI. This nomogram was developed with six perioperative predictors. Find each predictor’s point on the uppermost point scale and add them up. The total point projected to the bottom scale indicates the % probability of postoperative AKI. Abbreviations: AKI, Acute kidney injury; CKD, Chronic kidney disease; NSAIDs, Non-steroidal anti-inflammatory drugs

The training dataset from 599 patients was used to establish the predictive nomogram. The univariate logistic regression analysis of factors associated with postoperative AKI is summarized in Table 3. Multivariable logistic regression analysis demonstrated that the following variables were independent predictors of postoperative AKI in older patients with liver resection: age, history of CKD, hepatic inflow occlusion, intraoperative blood loss and transfusion, and perioperative use of NSAIDs (Table 4 and Additional file 2). These six predictors selected as the optimal subset for predicting postoperative AKI were incorporated into the predictive nomogram (Fig. 2). For an individual patient, find each of the six predictor’s points on the top line and add them together. The total points corresponding to the bottom line indicated the percentage of probability of postoperative AKI. An online calculator based on the multivariable logistic regression model was developed to allow clinicians to enter the values of the 6 predictors required for the risk score with automatic calculation of the likelihood (with 95% CI) that a patient will develop AKI (https://yuyao0505.shinyapps.io/DynNomapp/) (Additional file 3).

**Table 3:** Univariate logistic regression analysis of study variables vs postoperative AKI in the training cohort

Variables	***β*** Coefficient (95% CI)	OR (95% CI)	***P*** Value
Age, years	0.05 (0.01–0.10)	1.05 (1.01–1.10)	0.02
Sex, female vs male	0.11 (− 0.31–0.54)	1.12 (0.73–1.71)	0.61
BMI, kg·m^−2^	− 0.04 (− 0.10–0.03)	0.96 (0.90–1.03)	0.24
Hypertension, yes vs no	0.38 (− 0.03–0.80)	1.47 (0.97–2.22)	0.07
Diabetes, yes vs no	0.46 (− 0.03–0.96)	1.59 (0.97–2.61)	0.06
Cardiovascular diseases, yes vs no	0.11 (− 0.48–0.70)	1.11 (0.62–2.01)	0.72
CKD, yes vs no	1.07 (0.25–1.89)	2.92 (1.29–6.62)	0.01
Pulmonary diseases, yes vs no	−0.22 (− 1.19–0.76)	0.80 (0.30–2.14)	0.66
Hepatitis/ cirrhosis, yes vs no	−0.15 (− 0.67–0.37)	0.86 (0.51–1.45)	0.58
ASA PS, III-IV vs I-II	0.54 (0.05–1.03)	1.72 (1.05–2.81)	0.03
Pathology, vs hepatoma			
Cholangiocarcinoma	0.25 (− 0.32–0.82)	1.28 (0.72–2.27)	0.40
Hepatic Metastasis	0.35 (− 0.81–1.51)	1.41 (0.44–4.51)	0.56
Benign	−0.48 (− 1.08–0.12)	0.62 (0.34–1.12)	0.12
Hemoglobin, g·L^− 1^	−0.02 (− 0.03 - -0.01)	0.98 (0.97–0.99)	< 0.01
Albumin, g·L^− 1^	− 0.08 (− 0.13 - -0.03)	0.92 (0.88–0.97)	< 0.01
Total bilirubin, per 10 μmol·L^− 1^	0.04 (0.002–0.07)	1.04 (1.00–1.07)	0.04
Direct bilirubin, per 10 μmol·L^− 1^	0.05 (0.01–0.09)	1.05 (1.01–1.09)	0.02
Fasting blood glucose, μmol·L^− 1^	0.07 (− 0.02–0.16)	1.07 (0.98–1.17)	0.12
Creatinine, μmol·L^− 1^	0.004 (− 0.01–0.01)	1.00 (0.99–1.01)	0.39
eGFR, ml·min·1.73 m^− 2^	− 0.01 (− 0.02–0.01)	0.99 (0.98–1.01)	0.33
NSAIDs, yes vs no	1.54 (0.36–2.72)	4.67 (1.43–15.19)	0.01
Preoperative diuretics, yes vs no	0.52 (− 0.20–1.24)	1.68 (0.81–3.46)	0.16
Nephrotoxic antibiotics, yes vs no	0.54 (−0.11–1.19)	1.71 (0.89–3.28)	0.11
Glucocorticoid, vs None			
Dexamethasone	−0.20 (− 0.78–0.37)	0.82 (0.46–1.45)	0.49
Methylprednisolone	0.06 (− 0.43–0.56)	1.06 (0.65–1.75)	0.80
Vasoactive agents, vs none			
Hypertensive agents	0.48 (0.01–0.95)	1.62 (1.01–2.58)	0.04
Hypotensive agents	−0.57 (− 1.46–0.32)	0.56 (0.23–1.38)	0.21
Both	0.87 (0.17–1.56)	2.38 (1.18–4.78)	0.01
Intraoperative diuretic, vs no	0.57 (0.05–1.09)	1.76 (1.05–2.97)	0.03
MAP < 60 mmHg, yes vs no	0.19 (−0.24–0.63)	1.21 (0.78–1.87)	0.39
Duration of MAP < 60 mmHg, min	0.01 (0–0.02)	1.01 (1.00–1.02)	0.05
Duration of operation, h	0.26 (0.13–0.39)	1.30 (1.14–1.48)	< 0.01
Fluid balance, ml· kg^− 1^· h^− 1^	− 0.002 (− 0.05–0.04)	1.00 (0.95–1.04)	0.91
Hydroxyethyl starch, ml· kg^− 1^· h^− 1^	0.07 (− 0.03–0.18)	1.07 (0.97–1.19)	0.19
Ringer’s solution, ml· kg^− 1^· h^− 1^	− 0.02 (− 0.07–0.03)	0.98 (0.93–1.03)	0.47
Urine output, ml· kg^− 1^· h^− 1^	0.01 (− 0.11–0.13)	1.01 (0.90–1.14)	0.85
Blood loss, per 100 ml	0.10 (0.06–0.14)	1.10 (1.06–1.15)	< 0.01
Blood transfusion, yes vs no	1.23 (0.79–1.67)	3.41 (2.20–5.30)	< 0.01
Resection extent, vs right liver			
Left liver	−1.44 (−2.09 - -0.80)	0.23 (0.12–0.45)	< 0.01
Partial	− 1.08 (− 1.60 - -0.56)	0.34 (0.20–0.57)	< 0.01
Hepatic inflow occlusion, yes vs no	0.48 (0.04–0.92)	1.61 (1.04–2.51)	0.03
Duration of occlusion, min	0.01 (− 0.001–0.02)	1.01 (1.00–1.02)	0.12

*Abbreviations*: *AKI* Acute kidney injury, *ASA PS* American Society of Anesthesiologists physical score, *BMI* Body mass index, *CI* Confidential interval, *CKD* Chronic kidney disease, *eGFR* estimated glomerular filtration rate, *LOS* Length of hospital stay, *MAP* Mean arterial pressure, *NSAIDs* Non-steroidal anti-inflammatory drugs; OR, Odds ratio

**Table 4:** Predictors for postoperative AKI after liver resection in final multivariable logistic regression model

Intercept and variables	***β*** Coefficient (95% CI)	OR (95% CI)	***P*** Value
Intercept	−8.05 (− 11.75 - -4.36)	–	–
Age, years	0.06 (0.01–0.11)	1.06 (1.01–1.11)	0.01
Presence of CKD	0.93 (0.06–1.79)	2.52 (1.04–6.00)	0.04
Use of NSAIDs	1.27 (0.06–2.47)	3.55 (1.07–11.79)	0.04
Hepatic inflow occlusion	0.49 (0.02–0.96)	1.63 (1.02–2.60)	0.04
Blood loss, per 100 ml	0.06 (0.02–0.11)	1.07 (1.02–1.12)	0.01
Blood transfusion	0.79 (0.24–1.33)	2.20 (1.28–3.80)	< 0.01
Area under ROC curve (AUC-ROC)			
Training cohort: 0.73 (0.68–0.78)			
Validation cohort: 0.71 (0.63–0.80)			

*Abbreviations*: *AKI* Acute kidney injury, *AUC* Area under ROC curve, *CI* Confidential interval, *CKD* Chronic kidney disease, *NSAIDs* Non-steroidal anti-inflammatory drugs, *OR* Odds ratio, *ROC* Receiver operating characteristic

In addition, there were also results showing that univariate risk of intraoperative diuretics and vasopressors is statistically significant; while the multivariable model risk did not show statistical significance (Additional file 4). And no statistical significance of urine output and fluid balance were found in uni- and multivariable analyses (Table 3 and Additional file 4).

### Validation of the nomogram

**Fig. 3:**
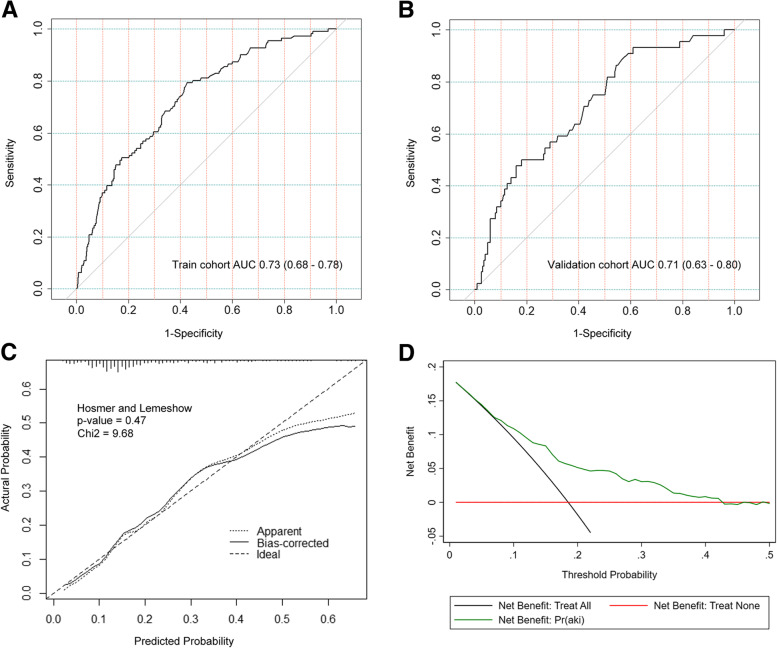
Validation of the nomogram: (**A**) ROC curve in the training dataset; (**B**) ROC curve in the validation dataset; (**C**) Calibration curve for the training dataset; (**D**) Decision curve analysis for the training dataset. Abbreviations: ROC, Receiver operating characteristic

The validation dataset of the remaining 244 patients was used to evaluate the model’s predictive performance. The nomogram had a satisfactory capacity with the areas under ROC curve (AUC) of 0.73 (95% confidential interval (CI): 0.68–0.78) and 0.71 (95% CI: 0.63–0.80) in the training and validation cohorts, respectively (Fig. 3A and B). Besides, the nomogram had a well-calibrated performance with Hosmer-Lemeshow chi-square value of 9.68 (*P* = 0.47) (Fig. 3C). The DCA showed the satisfactory net benefit that the patient could receive from the predictive nomogram, with a wide range (10 to 40%) of high-risk threshold (Fig. 3D).

### Summary risk score model

**Fig. 4:**
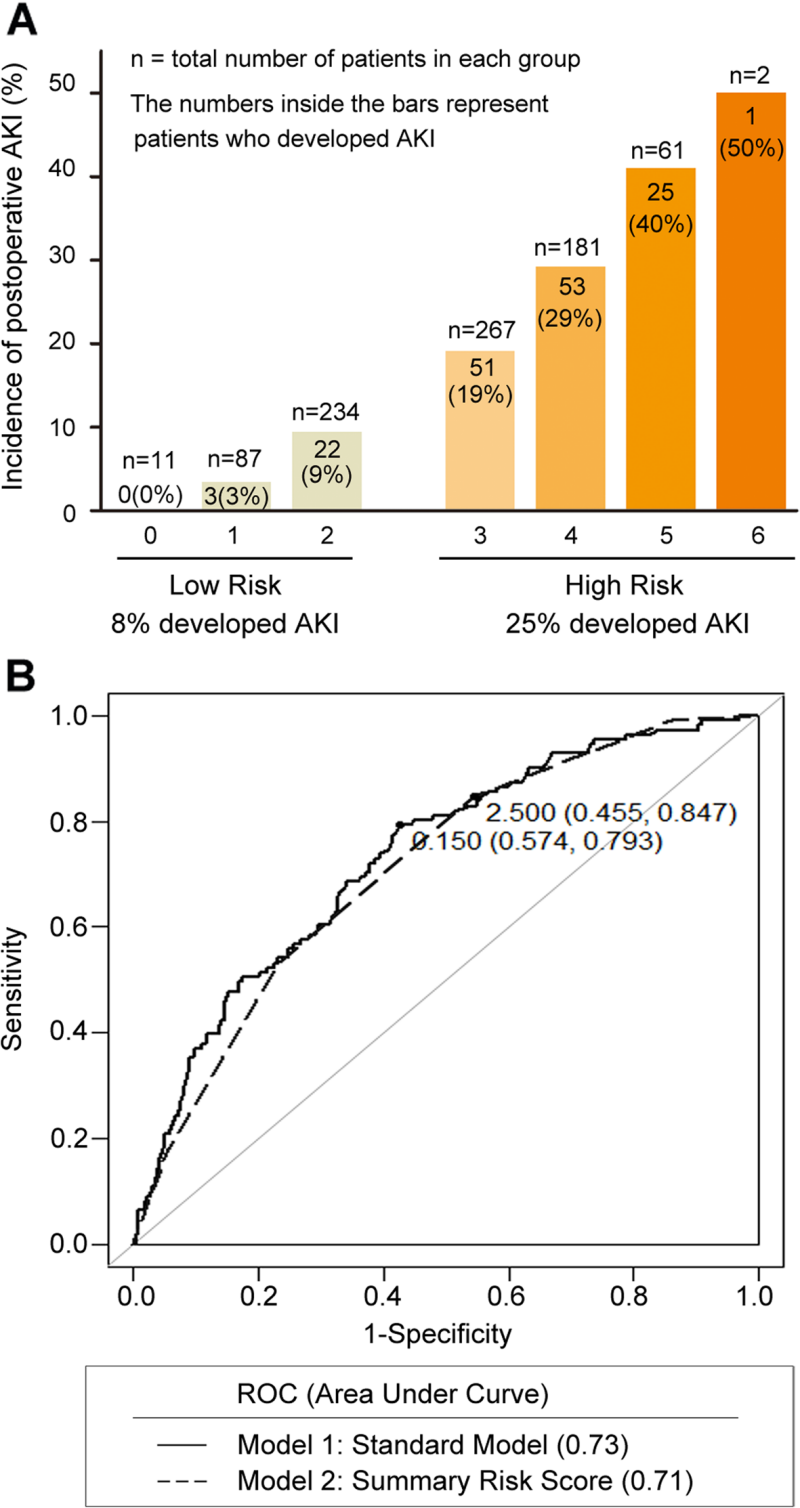
**A** The summary risk score model for predicting postoperative AKI. One point is assigned to each of the six predictors: age above 67 years, CKD, use of NSAIDs, intraoperative hepatic inflow occlusion, intraoperative blood loss > 300 mL, and blood transfusion. **B** Comparison between AUC-ROC of the multivariable logistic regression model and summary risk score model. Abbreviations: AKI, Acute kidney injury; AUC, Area under the ROC curve; CKD, Chronic kidney disease; NSAIDs, Non-steroidal anti-inflammatory drugs; ROC, Receiver operating characteristic

To promote the clinical application of the nomogram, variables selected for the predictive model were dichotomized and counted. Continuous parameters were divided into two subgroups with good or poor prediction based on the cut-off values determined by ROC analyses. One point was assigned to each of the following predictors: age above 67 years, CKD history, NSAIDs medication, intraoperative hepatic inflow occlusion, intraoperative blood loss > 300 mL, and requirement for blood transfusion (Additional files 5 and 6). The summary score for individual patients ranged from 0 to 6. The risk of AKI ranged from 4% with a score of 1, to 50%, with a score of 6. The summary scores were then grouped as low (0 to 2 points) and high-risk groups (3 to 6 points), as shown in Fig. 4A. AUC for predicting postoperative AKI was 0.71 (95% CI: 0.66–0.76) for the summary score model, which was not significantly different from the previous model (Fig. 4B).

## Discussion

Overall, our studies established a predictive nomogram and a simple risk score assessment to identify the risk of developing postoperative AKI in older patients post liver resection. Comprehensive information from overall 843 patients with hepatic resection over the past eight years was thoroughly reviewed and analyzed. The present nomogram and risk score assessment approach a landmark toward identifying strategies to reduce the prevalence of AKI in older patients following liver resection surgery.

### Summary of key findings

Our study summarized the incidence rates, predictors, and mortality impact of AKI in an older population following liver resection surgery. Our findings further verified that the morbidity of postoperative AKI was high (18.4%) among older populations, and associated with six-fold increased mortality rates compared with no occurrence of AKI, corroborating results from previous studies by Lim and Slankamenac [3, 22]. Therefore, a predictive nomogram and a simple risk score scale for postoperative AKI were developed and validated with six predictors readily available in clinical situations. The general overview of the main highlights of this study is as follows. First, we reviewed all relevant and eligible cases, acquired a few missing pieces of information. The large sample cohort allowed the development and validation of a predictive model. Second, the estimated GFR was selected as a better predictor of outcome to define preexisting renal dysfunction instead of serum creatinine level. Third, the simple summary risk score could be used to rapidly identify patients at high risk of developing AKI at the bedside without complex computation. Depending on this approach, we can help optimize decision-making in predicting and preventing postoperative renal dysfunction, and thus improve patients’ short-and long-term outcomes.

### Factors affecting the model

Several prediction models for postoperative AKI have been developed in noncardiac surgeries [4, 22, 23]. Although the occurrence of AKI remained high in about 20% of older patients following liver surgeries, the prediction of AKI in this subgroup population had been rarely concerned. Since postoperative AKI prediction can be affected by multiple factors resulting from the interactions between surgery, anesthesia procedure, and intensive care, therefore, therapeutic options to prevent and treat AKI following liver resection should involve pre-, intra-, and postoperative measures. By exploring risk factors for postoperative AKI after liver resection, we established a predictive nomogram for identifying high-risk patients that may need special attention after hepatic resection, so as to facilitate the clinical practice of early identification of such groups. Predictors identified in the present study included age, presence of CKD, NSAIDs medication, intraoperative hepatic inflow occlusion, the volume of blood loss, and transfusion. Thus, optimization of controllable factors such as preoperative renal dysfunction, NSAIDs usage, and blood transfusion due to substantial surgical bleeding may benefit patient’s survival outcomes. Surgical variables such as intraoperative hepatic occlusion and blood loss remained significant predictors of postoperative AKI. A possible explanation could be the complexity of the procedures needed for hepatic inflow occlusion, the requirement of extensive hepatic excision, and severe blood loss [24, 25].

In this context, minimization of surgical trauma and bleeding were fundamental protective strategies for renal conservation and also equally crucial to restore liver function and minimize resection extent. Inflammatory responses to surgical stress and trauma lead to tubular injury and subsequent development of AKI, potentially due to microcirculatory dysfunction, oxidative stress, and endothelial cell injury [26]. This group of patients should be given special attention because of their exceptionally high-risk exposure to perioperative procedures. Reducing the duration of hepatic occlusion has been shown to be favorable to control trauma and alleviate ischemia-reperfusion injury [27], which might contribute to the risk of renal injury mediated by inflammatory mediators and microcirculatory dysfunction [28].

Previous studies have suggested CKD as the most important patient-related risk factor [11]. It was not a new finding regarding the associations between CKD and postoperative AKI and death. While Chaudery et al has illustrated that in patients with a history of CKD, low estimated GFR does not markedly increase the mortality in the absence of AKI [8], highlighting AKI is likely to be a pivotal event connecting preoperative CKD and survival outcomes. In addition, NSAIDs inhibit cyclooxygenase activity and reduce prostaglandin secretion, resulting in tubular toxicity, renal vasoconstriction, decreasing renal blood flow, and low GFR [29, 30]. A systemic review conducted by Lee et al. has demonstrated that NSAIDs may cause a clinically transient reduction in renal function in the early postoperative period in patients with normal preoperative renal function [31]. Therefore, it is reasonable to minimize the nephrotoxic NSAID exposure in older patients concerning postoperative renal function, especially in the case of those with persistent renal dysfunction.

### Factors optimizing the model

Low central venous pressure (CVP) was required during the hepatectomy to reduce bleeding complications and mortality. Hughes has consolidated reduced blood loss and transfusion requirement by maintaining CVP below 5 mmHg, by intravenous fluid restriction, and by applying diuretics [32]. At the same time, the resultant hypovolemia and oliguria may increase the risks of renal impairment, as demonstrated by Myles in a study comparing restrictive and liberal fluid administration [5]. Historically, whether restrictive or liberal fluid therapy benefits outcomes remained controversial. Surgeons expect a very low CVP, while anesthesiologists argue against the low fluid load. The prolonged fluid restriction may lead to possible renal hypoperfusion with resultant oliguria. Vasopressors to correct hypovolemia could further aggravate oliguria. Therefore, the urine output criterion may result in an overestimation of AKI [33]. It has been suggested that intraoperative urine output of 0.3 ml·kg^− 1^·h^− 1^ was the optimal threshold for oliguria, nevertheless a moderate association and poor predictive value for postoperative AKI [34–36]. Numerous studies have established the negative bearing of diuretics usage in AKI in major abdominal surgeries [37]. In the present study, we found no significant difference in urine output between patients with and without AKI; postoperative AKI was associated with more employment of diuretics, which however did not increase the model’s predictive ability. The relationship between the predictive value of diuretics and urine output in AKI remained to discuss, and further studies are needed to confirm the associations, so as to promote potential targets for optimizing renal function in prospective clinical trials.

### Study limitations

There are some limitations in this study. First, the study aimed to evaluate the independent risk factors predicting postoperative AKI in older patients with liver resection. Therefore, the predictors involved were commonly available in clinical settings and did not include complex frailty and comorbidity index, which represent the risk associated with perioperative mortality. Second, although intraoperative factors affecting renal function were incorporated into the model, the target mean arterial pressure (MAP) during surgery and fluid management strategies were not available in this retrospective analysis, the surrogate indicators did not obviously increase the model’s predictive performance. This special attention will require further study and in combination with emerging biomarkers and novel oxygenation monitoring, clinical risk predictive models may aid in future investigations of effective prevention and treatment strategies. Third, data were derived from a retrospective single-center cohort. The reproducibility and generalization of the model in other populations are unknown. Before the model is implemented in clinical applications, it is essential to be externally validated in open, prospective multicenter studies.

## Conclusions

A nomogram and risk scoring system that accurately predicts postoperative AKI in older patients undergoing liver resection were established in this study. This model considered AKI based on six conveniently available variables in clinical conditions. It could provide guidance for both clinical decision-making and scheduling a tighter perioperative follow-up in AKI high-risk patients.

## Supplementary Information

**Additional file 1.** Patient characteristics and perioperative variables stratified by AKI. Notes: “Vasopressors” in vasoactive agents include ephedrine, epinephrine, dopamine, norepinephrine, and phenylephrine. “Vasodilators” in vasoactive agents include urapidil and nicardipine. Nephrotoxic antibiotics refers to aminoglycoside and sulfonamide antibiotics. Continuous data are shown as medians (quartiles) and compared using Wilcoxon rank-sum test. Categorical variables are shown as frequencies (percentages) and compared using chi-squared test or Fisher’s exact test as appropriate. Abbreviations: AKI, Acute kidney injury; ASA PS: American Society of Anesthesiologists physical score; BMI, Body mass index; CI, Confidential interval; CKD, Chronic kidney disease; eGFR, estimated glomerular filtration rate; LOS, Length of hospital stay; MAP, Mean arterial pressure; NSAIDs, Non-steroidal anti-inflammatory drugs; OR, Odds ratio.

**Additional file 2.** Graphical representation of the regression model with confidence intervals. Abbreviations: NSAIDs, Non-steroidal anti-inflammatory drugs.

**Additional file 3.** The online web-based calculator for predicting acute kidney injury among older patients with liver resection surgery. https://yuyao0505.shinyapps.io/DynNomapp. Abbreviations: CKD, Chronic kidney disease; NSAIDs, Non-steroidal anti-inflammatory drugs.

**Additional file 4.** Predictors for AKI in multivariable logistic regression model including intraoperative diuretics, urine output, vasopressors, and fluid balance. Notes: “Vasopressors” in vasoactive agents include ephedrine, epinephrine, dopamine, norepinephrine, and phenylephrine. Abbreviations: CI, Confidential interval; CKD, Chronic kidney disease; NSAIDs, Non-steroidal anti-inflammatory drugs; OR, Odds ratio.

**Additional file 5.** Summary risk score model and sensitivity/specificity values of the predictors for AKI. Abbreviations: AUC, Area under ROC curve; CKD, Chronic kidney disease; NSAIDs, Non-steroidal anti-inflammatory drugs; ROC, Receiver operating characteristic.

**Additional file 6.** Sensitivity and specificity values of risk score model.

## Data Availability

The datasets analyzed during the current study are available from the corresponding author on reasonable request.

## Abbreviations

AKI: Acute kidney injury
ASA PS: American Society of Anesthesiologists physical score
AUC: Areas under the ROC curve
CKD: Chronic kidney disease
CKD-EPI: CKD Epidemiology Collaboration
CVP: Central venous pressure
DCA: Decision curve analysis
eGFR: estimated glomerular filtration rate
EMR: Electronic medical record
EPV: Events per variable
LOS: Length of hospital stay
MAP: Mean arterial pressure
KDIGO: Kidney Disease: Improving Global Outcomes
NSAIDs: Non-steroidal anti-inflammatory drugs
ROC: Receiver operating characteristic
TRIPOD: Transparent Reporting of a Multivariable Prediction Model for Individual Prognosis or Diagnosis
