# Supplementary material for: Development and validation of a risk nomogram for postoperative acute kidney injury in older patients undergoing liver resection: a pilot study
Source: BMC Anesthesiol. 2022 Jan 13;22:22. doi: 10.1186/s12871-022-01566-z (PMC8756684; doi:10.1186/s12871-022-01566-z)
Supplement: Supplementary file 1 — Additional file 1. Patient characteristics and perioperative variables stratified by AKI. Notes: “Vasopressors” in vasoactive agents include ephedrine, epinephrine, dopamine, norepinephrine, and phenylephrine. “Vasodilators” in vasoactive agents include urapidil and nicardipine. Nephrotoxic antibiotics refers to aminoglycoside and sulfonamide antibiotics. Continuous data are shown as medians (quartiles) and compared using Wilcoxon rank-sum test. Categorical variables are shown as frequencies (percentages) and compared using chi-squared test or Fisher’s exact test as appropriate. Abbreviations: AKI, Acute kidney injury; ASA PS: American Society of Anesthesiologists physical score; BMI, Body mass index; CI, Confidential interval; CKD, Chronic kidney disease; eGFR, estimated glomerular filtration rate; LOS, Length of hospital stay; MAP, Mean arterial pressure; NSAIDs, Non-steroidal anti-inflammatory drugs; OR, Odds ratio. [file 12871_2022_1566_MOESM1_ESM.docx]

**Additional file 1**

Patient characteristics and perioperative variables stratified by AKI.

| **Variables** | **Without AKI**  **(n=688)** | **With AKI**  **(n=155)** | ***P* Value** |
| --- | --- | --- | --- |
| Age, years | 68 (66, 72) | 69 (67, 73) | 0.02 |
| Sex (Male), n (%) | 454 (66.0) | 99 (63.9) | 0.64 |
| BMI, kg·m^-2^ | 23.7 (21.6, 25.9) | 23.1 (20.7, 25.9) | 0.09 |
| Hypertension, n (%) | 251 (36.5) | 68 (43.9) | 0.10 |
| Diabetes, n (%) | 120 (17.4) | 37 (23.9) | 0.07 |
| Cardiovascular diseases, n (%) | 100 (14.5) | 23 (14.8) | 0.90 |
| CKD, n (%) | 19 (2.8) | 13 (8.4) | < 0.01 |
| Pulmonary diseases, n (%) | 37 (5.4) | 7 (4.5) | 0.84 |
| Hepatitis/ cirrhosis, n (%) | 141 (20.5) | 33 (21.3) | 0.83 |
| ASA PS, n (%)  I-II  III-IV | 575 (83.6)  113 (16.4) | 119 (76.8)  36 (23.2) | 0.05 |
| Pathology, n (%)  Hepatoma  Cholangiocarcinoma  Hepatic Metastasis  Benign | 437 (63.5)  97 (14.1)  19 (2.8)  135 (19.6) | 100 (64.5)  31 (20.0)  5 (3.2)  19 (12.3) | 0.07 |
| Hemoglobin, g·L^-1^ | 132 (122, 142) | 125 (113, 138) | < 0.01 |
| Albumin, g·L^-1^ | 39.2 (36.6, 41.7) | 37.8 (34.8, 40.8) | < 0.01 |
| Total bilirubin, μmol·L^-1^ | 12.8 (9.3, 18.6) | 13.3 (9.3, 28.8) | 0.08 |
| Direct bilirubin, μmol·L^-1^ | 4.2 (3.0, 7.1) | 5.4 (3.2, 18.9) | < 0.01 |
| Fasting blood glucose, μmol·L^-1^ | 5.03 (4.58, 5.76) | 5.20 (4.58, 6.41) | 0.04 |
| Creatinine, μmol·L^-1^ | 68.1 (59.0, 78.8) | 67.2 (56.7, 78.9) | 0.50 |
| eGFR, ml·min·1.73 m^-2^ | 89.8 (82.1, 94.5) | 90.0 (79.7, 96.0) | 0.96 |
| NSAIDs, n (%) | 606 (88.1) | 151 (97.4) | < 0.01 |
| Preoperative diuretics, n (%) | 43 (6.3) | 17 (11.0) | 0.06 |
| Nephrotoxic antibiotics, n (%) | 62 (9.0) | 17 (11.0) | 0.45 |
| Glucocorticoid, n (%)  Dexamethasone  Methylprednisolone  None | 196 (28.5)  309 (44.9)  183 (26.6) | 38 (24.5)  74 (47.7)  43 (27.7) | 0.61 |
| Vasoactive agents, n (%)  None  Vasopressors  Vasodilators  Both | 378 (54.9)  185 (26.9)  84 (12.2)  41 (6.0) | 70 (45.2)  60 (38.7)  10 (6.5)  15 (9.7) | < 0.01 |
| Intraoperative diuretics, n (%) | 97 (14.1) | 41 (26.5) | <0.01 |
| MAP < 60 mmHg, n (%) | 436 (63.4) | 111 (71.6) | 0.06 |
| Duration of MAP < 60 mmHg, min | 5 (0, 15) | 10 (0, 25) | 0.01 |
| Duration of operation, h | 3.2 (2.5, 4.3) | 4.0 (3.0, 5.2) | < 0.01 |
| Fluid balance, ml·kg^-1^·h^-1^ | 11.8 (9.2, 14.8) | 11.2 (8.4, 15) | 0.36 |
| Hydroxyethyl starch, ml·kg^-1^·h^-1^ | 3.6 (2.6, 4.9) | 3.9 (2.7, 4.9) | 0.14 |
| Ringer's solution, ml·kg^-1^·h^-1^ | 10.0 (7.7, 12.9) | 9.3 (6.9, 12.7) | 0.06 |
| Urine output, ml·kg^-1^·h^-1^ | 1.7 (0.9, 2.9) | 1.8 (1.2, 2.7) | 0.31 |
| Blood loss, 100 ml | 3 (2, 5) | 5 (3, 8) | < 0.01 |
| Blood transfusion, n (%) | 125 (18.2) | 67 (43.2) | < 0.01 |
| Resection extent, n (%)  Right liver  Left liver  Partial | 82 (11.9)  195 (28.3)  417 (59.7) | 38 (24.5)  32 (20.6)  85 (54.8) | < 0.01 |
| Hepatic inflow occlusion, n (%) | 406 (59.0) | 107 (69.0) | 0.02 |
| Duration of occlusion, min | 13 (0, 29) | 15 (0, 30) | 0.07 |
| LOS, days | 10 (8,13) | 11 (9,16) | < 0.01 |
| Death before discharge, n (%) | 5 (0.7) | 8 (5.2) | < 0.01 |

**Notes:** “Vasopressors” in vasoactive agents include ephedrine, epinephrine, dopamine, norepinephrine, and phenylephrine. “Vasodilators” in vasoactive agents include urapidil and nicardipine. Nephrotoxic antibiotics refers to aminoglycoside and sulfonamide antibiotics. Continuous data are shown as medians (quartiles) and compared using Wilcoxon rank-sum test. Categorical variables are shown as frequencies (percentages) and compared using chi-squared test or Fisher’s exact test as appropriate.

**Abbreviations:** AKI, Acute kidney injury; ASA PS: American Society of Anesthesiologists physical score; BMI, Body mass index; CI, Confidential interval; CKD, Chronic kidney disease; eGFR, estimated glomerular filtration rate; LOS, Length of hospital stay; MAP, Mean arterial pressure; NSAIDs, Non-steroidal anti-inflammatory drugs; OR, Odds ratio.
