# Supplementary material for: Development and validation of a risk nomogram for postoperative acute kidney injury in older patients undergoing liver resection: a pilot study
Source: BMC Anesthesiol. 2022 Jan 13;22:22. doi: 10.1186/s12871-022-01566-z (PMC8756684; doi:10.1186/s12871-022-01566-z)
Supplement: Supplementary file 2 — Additional file 2. Graphical representation of the regression model with confidence intervals. Abbreviations: NSAIDs, Non-steroidal anti-inflammatory drugs. [file 12871_2022_1566_MOESM2_ESM.docx]

**Additional file 2**

Graphical representation of the regression model with confidence intervals.

**
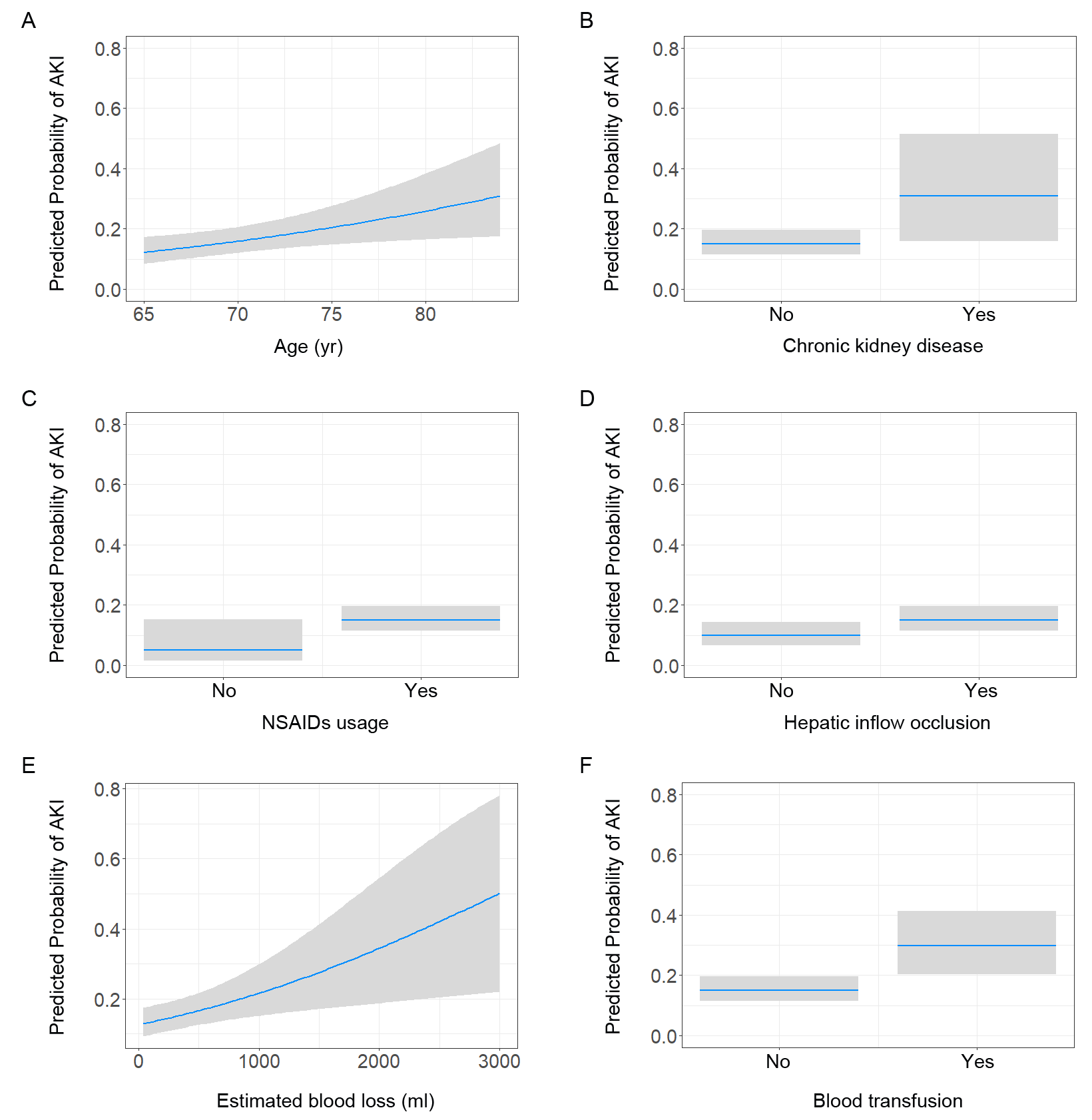
**

**Abbreviations:** NSAIDs, Non-steroidal anti-inflammatory drugs.
