# Supplementary material for: Development and validation of a risk nomogram for postoperative acute kidney injury in older patients undergoing liver resection: a pilot study
Source: BMC Anesthesiol. 2022 Jan 13;22:22. doi: 10.1186/s12871-022-01566-z (PMC8756684; doi:10.1186/s12871-022-01566-z)
Supplement: Supplementary file 3 — Additional file 3. The online web-based calculator for predicting acute kidney injury among older patients with liver resection surgery. https://yuyao0505.shinyapps.io/DynNomapp. Abbreviations: CKD, Chronic kidney disease; NSAIDs, Non-steroidal anti-inflammatory drugs. [file 12871_2022_1566_MOESM3_ESM.docx]

**Additional file 3**

The online web-based calculator for predicting acute kidney injury among older patients with liver resection surgery. <https://yuyao0505.shinyapps.io/DynNomapp>.


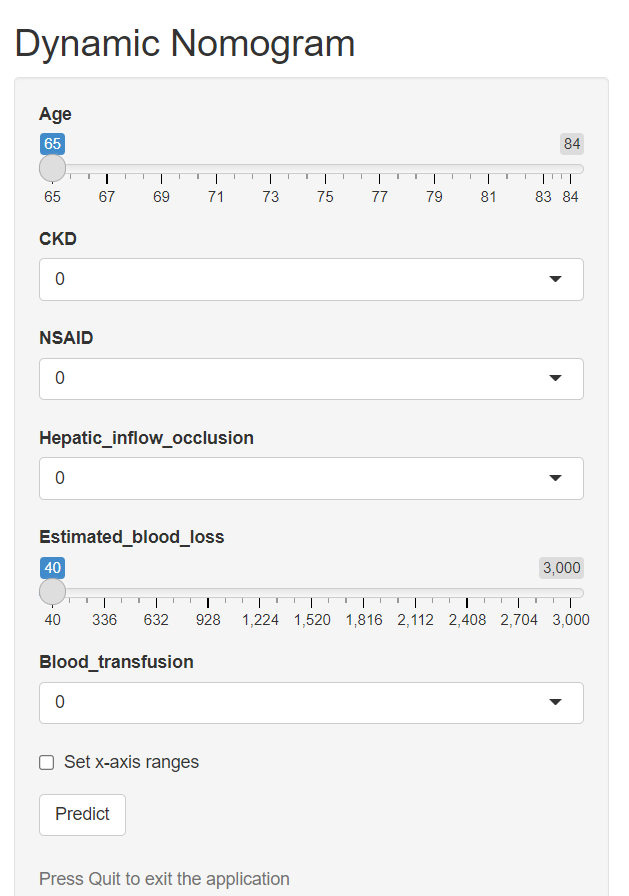


**Abbreviations:** CKD, Chronic kidney disease; NSAIDs, Non-steroidal anti-inflammatory drugs.
