# Supplementary material for: Development and validation of a risk nomogram for postoperative acute kidney injury in older patients undergoing liver resection: a pilot study
Source: BMC Anesthesiol. 2022 Jan 13;22:22. doi: 10.1186/s12871-022-01566-z (PMC8756684; doi:10.1186/s12871-022-01566-z)
Supplement: Supplementary file 4 — Additional file 4. Predictors for AKI in multivariable logistic regression model including intraoperative diuretics, urine output, vasopressors, and fluid balance. Notes: “Vasopressors” in vasoactive agents include ephedrine, epinephrine, dopamine, norepinephrine, and phenylephrine. Abbreviations: CI, Confidential interval; CKD, Chronic kidney disease; NSAIDs, Non-steroidal anti-inflammatory drugs; OR, Odds ratio. [file 12871_2022_1566_MOESM4_ESM.docx]

**Additional file 4**

Predictors for AKI in multivariable logistic regression model including intraoperative diuretics, urine output, vasopressors, and fluid balance.

| **Intercept and variables** | ***β* Coefficient (95% CI)** | **OR (95% CI)** | ***P* Value** |
| --- | --- | --- | --- |
| Intercept | -8.54 (-12.12 – -4.38) | — | — |
| Age, years | 0.06 (0.01 – 0.11) | 1.06 (1.01 – 1.12) | 0.01 |
| CKD | 0.95 (0.04 – 1.82) | 2.60 (1.04 – 6.20) | 0.03 |
| Use of NSAIDs | 1.34 (0.28 – 2.79) | 3.82 (1.32 – 16.28) | 0.03 |
| Hepatic inflow occlusion | 0.48 (0.01 – 0.96) | 1.62 (1.01 – 2.62) | 0.05 |
| Blood loss, per 100 ml | 0.06 (0.01 – 0.11) | 1.06 (1.01 – 1.11) | 0.02 |
| Blood transfusion | 0.77 (0.20 – 1.32) | 2.15 (1.22 – 3.76) | 0.01 |
| Intraoperative diuretics | 0.23 (-0.37 – 0.79) | 1.25 (0.69 – 2.22) | 0.44 |
| Vasopressor | 0.28 (-0.20 – 0.75) | 1.32 (0.82 – 2.13) | 0.25 |
| Urine output (ml· kg^-1^· h^-1^) | 0.04 (-0.09 – 0.17) | 1.04 (0.91 – 1.18) | 0.53 |
| Fluid balance (ml· kg^-1^· h^-1^) | 0.02 (-0.03 – 0.07) | 1.02 (0.97 – 1.07) | 0.41 |

**Notes:** “Vasopressors” in vasoactive agents include ephedrine, epinephrine, dopamine, norepinephrine, and phenylephrine.

**Abbreviations:** CI, Confidential interval; CKD, Chronic kidney disease; NSAIDs, Non-steroidal anti-inflammatory drugs; OR, Odds ratio.
