# Supplementary material for: Development and validation of a risk nomogram for postoperative acute kidney injury in older patients undergoing liver resection: a pilot study
Source: BMC Anesthesiol. 2022 Jan 13;22:22. doi: 10.1186/s12871-022-01566-z (PMC8756684; doi:10.1186/s12871-022-01566-z)
Supplement: Supplementary file 5 — Additional file 5. Summary risk score model and sensitivity/specificity values of the predictors for AKI. Abbreviations: AUC, Area under ROC curve; CKD, Chronic kidney disease; NSAIDs, Non-steroidal anti-inflammatory drugs; ROC, Receiver operating characteristic. [file 12871_2022_1566_MOESM5_ESM.docx]

**Additional file 5**

Summary risk score model and sensitivity/specificity values of the predictors for AKI.

| **Predictors** | **Score** | | **Sensitivity** | **Specificity** | **AUC-ROC** |
| --- | --- | --- | --- | --- | --- |
|  | **0** | **1** |  |  |  |
| Age | ≤67 yr | >67 yr | 0.68 | 0.43 | 0.57 |
| CKD | no | yes | 0.09 | 0.97 | 0.53 |
| Use of NSAIDs | no | yes | 0.97 | 0.11 | 0.54 |
| Hepatic inflow occlusion | no | yes | 0.69 | 0.42 | 0.55 |
| Blood loss | ≤300 ml | >300 ml | 0.69 | 0.57 | 0.67 |
| Blood transfusion | no | yes | 0.43 | 0.82 | 0.63 |

**Abbreviations:** AUC, Area under ROC curve; CKD, Chronic kidney disease; NSAIDs, Non-steroidal anti-inflammatory drugs; ROC, Receiver operating characteristic.
