# Supplementary material for: Development and validation of a risk nomogram for postoperative acute kidney injury in older patients undergoing liver resection: a pilot study
Source: BMC Anesthesiol. 2022 Jan 13;22:22. doi: 10.1186/s12871-022-01566-z (PMC8756684; doi:10.1186/s12871-022-01566-z)
Supplement: Supplementary file 6 — Additional file 6. Sensitivity and specificity values of risk score model. [file 12871_2022_1566_MOESM6_ESM.docx]

**Additional file 6**

Sensitivity and specificity values of risk score model.

| **AKI risk score** | **Sensitivity** | **Specificity** |
| --- | --- | --- |
| 0 | 1.00 | 0.02 |
| 1 | 0.99 | 0.14 |
| 2 | 0.85 | 0.46 |
| 3 | 0.53 | 0.77 |
| 4 | 0.18 | 0.94 |
| 5 | 0.01 | 1.00 |
| 6 | 0 | 0 |
